# Diet and physical activity behaviours among people with insomnia symptoms: a cross-sectional survey to understand treatment needs and acceptability

**DOI:** 10.1080/21642850.2026.2721023

**Published:** 2026-08-24

**Authors:** Jacy Hyland, Moira Junge, Joseph J. Scott, David Cunnington, Mia A. Schaumberg, Mathew J. Summers, Timothy Baird, Claire M. Ellender, Caitlin Fehily, Emma Axelsson, Sara Winter, Jenny Bowman, Hattie H. Wright, Christopher D. Askew, Alexandra P. Metse

**Affiliations:** a School of Health, University of the Sunshine Coast, Sippy Downs, QLD, Australia; b VasoActive Research Group, School of Health, University of the Sunshine Coast, Sippy Downs, Australia; c The Sleep Health Foundation, North Melbourne, VIC, North Melbourne, Australia; d School of Education and Tertiary Access, University of the Sunshine Coast, Sippy Downs, QLD, Australia; e School of Education, Edith Cowan University, Joondalup, WA, Australia; f Thompson Institute, University of the Sunshine Coast, Birtinya, QLD, Australia; g School of Human Movement and Nutrition Sciences, The University of Queensland, St Lucia, QLD, Australia; h Sunshine Coast University Hospital, Birtinya, QLD, Australia; i School of Medicine and Dentistry, Griffith University, Birtinya, QLD, Australia; j Centre for Bioinnovation, University of the Sunshine Coast, Sippy Downs, QLD, Australia; k Sunshine Coast Health Institute, Sunshine Coast Hospital and Health Service, Birtinya, QLD, Australia; l School of Medicine, University of Queensland, QLD, Brisbane, Australia; m School of Science, University of Newcastle, Callaghan, NSW, Australia; n Allied Health Research Collaborative, The Prince Charles Hospital, Chermside, QLD, Australia; o Hunter Medical Research Institute (Population Health Program), Newcastle, NSW, Australia

**Keywords:** Acceptability, behavioural sleep interventions, insomnia, Mediterranean diet, physical activity

## Abstract

**Background:**

High adherence to a Mediterranean diet and regular moderate-intensity physical activity (≥5 days/week, ≥30 minutes/day) reduce the risk of insomnia symptom onset and maintenance, yet engagement in these behaviours among adults with insomnia symptoms and the acceptability of lifestyle support are not well understood.

**Methods:**

Objectives: (1) characterise diet and physical activity behaviours among people with insomnia symptoms, including adherence to the Mediterranean diet and physical activity guidelines (moderate-intensity physical activity on ≥5days/week for ≥30 minutes/day), as well as the regularity of these behaviours; (2) explore clinical and socio-demographic factors associated with these behaviours; and (3) investigate the acceptability of receiving support to optimise diet and physical activity as part ofin insomnia treatment. Australian adults with insomnia symptoms completed an online cross-sectional survey (*n* = 308; 85% female; 18–65 years). Diet and physical activity were assessed using the Mediterranean Diet Adherence Screener (MEDAS) and the International Physical Activity Questionnaire (IPAQ), alongside the regularity of meal and activity patterns. Acceptability was measured using the Acceptability of Intervention Measure (AIM). Logistic regression explored associations with gender, age, insomnia severity, and education.

**Results:**

Most participants reported low Mediterranean diet adherence (72%), low or -moderate activity levels (66%), did not meet physical activity guidelines (69%), and had irregular meal (71%) and activity patterns (60%). Mediterranean diet adherence was lower in men, younger adults, and those with lower education; no factors were associated with physical activity. Support was acceptable to 57% (mean AIM = 3.8/5, SD = 0.8), with higher acceptability among women and those with severe insomnia symptoms.

**Conclusions:**

Findings provide preliminary evidence of an unmet need for interventions to support lifestyle behaviour change among those with insomnia symptoms. While support is acceptable to many, targeted approaches may be needed for subgroups with lower adherence and acceptability.

## 1. Introduction

Insomnia disorder is characterised by dissatisfaction with sleep quantity and quality, associated with difficulties initiating or maintaining sleep or early morning awakenings (American Psychiatric Association, 2022). Global estimates place the prevalence of clinically relevant insomnia at 16% of the adult population (Benjafield et al., 2025). However, notably larger proportions experience subthreshold insomnia symptoms. In Australia, for example, a representative sample of adults (*n* = 2044) found that almost 60% frequently (≥3 times per week) experience at least one insomnia symptom and 40% experience two or more symptoms (Reynolds et al., 2019).

Insomnia symptoms are associated with significant preventable morbidity and mortality, including an increased risk of cardiovascular diseases, depression, anxiety and Alzheimer's disease (Wu et al., 2023). Additionally, insomnia disorder poses a substantial economic burden, with estimated 2019 costs in high-income countries ranging from US$1.8 to $207.5 billion (Hafner et al., 2023). The high prevalence of insomnia disorder and symptoms, along with the significant burden, highlights the need for focused research attention and effective intervention.

Cognitive behavioural therapy for insomnia (CBT-I) is considered the gold standard for insomnia treatment. Although CBT-I is very effective for some, effectiveness declines over time (van der Zweerde et al., 2019), with only 50% of treatment *responders* achieving remission (Muench et al., 2022). One potential reason for the poor treatment response to CBT-I in some individuals could be the lack of routine inclusion of support to address influential lifestyle behaviours (De Pasquale et al., 2024). Indeed, recent insomnia treatment guidelines (2023) call for the development and evaluation of new therapeutic avenues, specifically noting lifestyle interventions such as those targeting physical activity levels, as a promising component of treatment (Riemann et al., 2024).

Poor diet and physical inactivity are significant risk factors for the onset and persistence of insomnia symptoms (Kline et al., 2021; Metse et al., 2021; Zuraikat et al., 2021). Systematic review evidence (*n* = 15 studies) indicates that increased physical activity in people with insomnia results in improvements to objective sleep parameters, including sleep efficiency, sleep onset latency, wake after sleep onset and total sleep time (Ferreira et al., 2023). A further review and meta-analysis of the effect of physical exercise interventions on insomnia (*n* = 19 RCTs) found significant improvements in objective and subjective sleep parameters (Riedel et al., 2024). Specifically, there is strong evidence for the benefit of at least 30 minutes of moderate-to-vigorous physical activity on five or more days per week (Riedel et al., 2024), with the duration, intensity and frequency in line with the minimum recommendations in Australian physical activity guidelines (Australian Government Department of Health Disability & Ageing, 2026). Additionally, systematic review evidence (*n* = 37 observational studies) suggests overall dietary patterns are associated with risk of insomnia symptoms (Arab et al., 2024). The Mediterranean diet, specifically, has received substantial research attention within the context of insomnia, potentially due to its well-established guidelines, validated measurements and extensive previous research across a range of health outcomes. Evidence from a scoping review of epidemiological studies (*n* = 17) suggests higher adherence to the Mediterranean diet is associated with reduced risk of insomnia symptoms across diverse populations (Scoditti et al., 2022). Additionally, evidence suggests that adherence to the Mediterranean diet may alleviate insomnia symptoms, as a systematic review and meta-analysis (*n* = 62 studies) found that following this diet was associated with improvements in sleep features such as sufficient sleep duration and good sleep quality (Arab et al., 2025). More specifically, scoring 9 or above on the Mediterranean Diet Adherence Screener (MEDAS) has been considered high adherence and associated with better sleep quality (Campanini et al., 2017). In addition, eating and physical activity patterns are zeitgebers, or environmental cues that synchronise the circadian rhythm to the 24-hour day, with regular patterns of meals and physical activity conducive to improved sleep quality (Haupt et al., 2021; Healy et al., 2021). As such, there is a need to consider the regularity of these behaviours among people experiencing insomnia symptoms.

The exact mechanisms by which diet and physical activity contribute to insomnia symptom reduction are still under investigation but may invovle their beneficial effects on inflammation (Scoditti et al., 2022; You et al., 2023), gut microbiota composition (Scoditti et al., 2022), melatonin biosynthesis (Patel & Cheung, 2025) and functioning of the default mode network (Li et al., 2017; Marques et al., 2015). Despite this, a precise understanding of these mechanisms is not needed for individuals with insomnia symptoms to experience the health benefits associated with improving diet and physical activity. There are likely therapeutic benefits in supporting people with insomnia to achieve 30 minutes of moderate-intensity physical activity 5 days a week, high adherence to the Mediterranean diet, and regular eating and physical activity patterns. However, the proportion who may benefit from treatment addressing these behaviours is unclear, as research characterising diet and physical activity among those with insomnia symptoms is limited.

One study explored diet and physical activity behaviours among people with poor sleep health including various insomnia symptoms (*n* = 1265) and found that 42% to 90% have a poor diet and/or are physically inactive (Metse et al., 2021). However, diet was assessed only briefly in terms of the number of serves of fruit and vegetables, rather than considering the overall diet pattern or extent of adherence to the Mediterranean diet. Further, physical activity was not measured in relation to physical activity guidelines, precluding the ability to assess against guideline-based thresholds associated with insomnia treatment benefits. In addition, the regularity of these behaviours was not considered, nor were subgroups of symptom severity examined for higher risk of poor lifestyle behaviours. Further research is required to explore the relationship between insomnia and adherence to physical activity guidelines and the Mediterranean diet, and whether diet and physical activity are acceptable intervention targets for people with insomnia symptoms.

‘Acceptability’ refers to the extent to which an intervention is perceived as agreeable, palatable and satisfactory (Proctor et al., 2011). This construct has been strongly associated with treatment adherence and outcomes across health disciplines (Sekhon et al., 2017). Ensuring an intervention is acceptable to target populations is an essential first step in the intervention development process (Proctor et al., 2011). In recent years, increasing attention has been given to the acceptability of variations of CBT-I (Zhang et al., 2022). However, to date, no research has explored the acceptability of receiving adequate support to optimise diet or physical activity as part of routine insomnia treatment.

This paper therefore aims to (1) characterise diet and physical activity behaviours among people with insomnia, including adherence to the Mediterranean diet and physical activity guidelines as well as the regularity of these behaviours; (2) explore clinical and socio-demographic factors associated with these behaviours; and (3) investigate the acceptability of receiving support to optimise diet and physical activity in the treatment of insomnia.

## 2. Methods

### 2.1. Study design and recruitment

A cross-sectional online survey was conducted using the Qualtrics platform (https://www.qualtrics.com).

Eligible participants were English-speaking adults aged 18–65 years, residing in Australia and reported at least subthreshold insomnia with at least mild issues initiating or maintaining sleep (i.e. scored ≥8 on the Insomnia Severity Index (Morin et al., 2011) and rated ≥1 across items 1 to 3, indicating more than non-restorative/daytime symptoms of insomnia). Participants were excluded if pregnant/lactating, reported risky alcohol use [AUDIT-C ≥3 (Bush et al., 1998)], obstructive sleep apnoea (symptoms or treatment), or a history of diagnosis/treatment for autism spectrum disorder, attention deficit hyperactivity disorder, narcolepsy, or dementia. Eligible participants were identified via a brief screening survey, and after providing informed consent, completed the full survey.

Participants were recruited from 1 May to 5 August 2024 via social media, university flyers, and an online recruitment platform ‘Prime Panels’, developed by CloudResearch. Prime Panels participants were offered monetary compensation; all other participants could enter a prize draw for one of five AU$100 prizes.

### 2.2. Measures

The survey was initially developed by the research team. It was then reviewed for content validity by clinicians and researchers in the field of sleep, psychology, exercise physiology and dietetics, and for clarity by a consumer group comprising individuals with lived experience of insomnia. Following feedback, minor wording changes were made throughout, and the survey was shortened by removing non-essential items.

#### 2.2.1. Socio-demographics

Socio-demographic information was gathered, including age, gender, education, Aboriginal and Torres Strait Islander status, country of birth, primary language spoken, employment status and marital status.

#### 2.2.2. Clinical information

Insomnia symptoms were measured using the Insomnia Severity Index (ISI), a validated 7-item questionnaire rated on a 5-point Likert scale (Morin et al., 2011). Responses were summed, with a higher score indicating greater insomnia severity (Morin et al., 2011). The ISI has previously demonstrated strong internal consistency (Cronbach's α of 0.90 and 0.91), adequate discriminatory capacity and significant convergent validity with a range of related constructs (Morin et al., 2011).

The self-rated health (SRH) item (Form 3) was used as a measure of general health status (Cullati et al., 2020). This is a validated single-item measure rated on a 5-point Likert scale where a lower score indicates better general health.

Participants responded to a purpose-developed item assessing the 12-month point prevalence of prior mental health diagnosis (response options: Yes, No, Prefer not to say). Those who responded ‘Yes’, indicated which diagnoses they had received from a list of mental health disorders (response options also included ‘Prefer not to say’ and ‘Other’ [open text box]).

Participants completed purpose-developed items on prior insomnia treatment (provider, treatment type and whether change to diet or physical activity was mentioned or supported).

#### 2.2.3. Diet

Mediterranean diet adherence was assessed using the 14-tem Mediterranean Diet Adherence Screener [MEDAS, (Martínez-González et al., 2012)]. Items are scored (0/1) and summed (0–14), with higher scores indicating greater adherence. MEDAS scores were categorised as low (≤5), moderate (6–8) and high (≥9).

Participants completed four items concerning meal pattern regularity for a typical workday and a typical weekend/non-work day, adapted from the Chrononutrition Profile Questionnaire (Veronda et al., 2020). Meal patterns were classified as irregular if the number of meals differed between workdays and non-workdays, or if the meal timing differed by more than one hour. This one-hour threshold was defined prior to analysis and selected to align with precedent in sleep regularity literature, where variation of more than one hour in sleep/wake timing is commonly used to assess regular sleep/wake timing, including in the RU-SATED sleep health measure (Ravyts et al., 2021). Participants reporting the same number of meals but answering ‘don't know’ or ‘not applicable’ to timing items were classified as ‘unsure’ and treated as missing for association analysis.

#### 2.2.4. Physical activity

The International Physical Activity Questionnaire-Short Form (IPAQ-SF) (Craig et al., 2017) assessed habitual physical activity over the past 7 days. Participants recalled and self-reported time spent in moderate and vigorous physical activity, walking and sitting over the previous week, reported as minutes/day, hours/day and days/week. Responses were converted to expended metabolic equivalent tasks (MET)-minutes (a measure which incorporates activity intensity and duration), using IPAQ scoring guidelines (Forde, 2018). Only values of 10 or more minutes were included; values <10 mins were recorded as zero. Values of >16 hours/day of moderate or vigorous activity were treated as outliers and excluded (Forde, 2018). Participants were then categorised as having low, moderate, or high physical activity per scoring guidelines.

Participants reported physical activity pattern regularity with the following purpose-developed item: ‘Do you follow a regular routine of physical activity? For example, this could include sport, exercise, swimming, dancing, housework, gardening, or yoga at a consistent time each day or week’. (Yes, No). Participants responding ‘No’ were classified as having an irregular physical activity pattern. Those responding ‘Yes’ reported the specific time they engage in physical activity; participants indicating ‘no regular time’ for this item were also classified as irregular.

#### 2.2.5. Acceptability

Acceptability of support to optimise diet and physical activity was assessed using the Acceptability of Intervention Measure [AIM, (Weiner et al., 2017)]. AIM is a validated 4-item measure rated on a 5-point Likert scale, where a higher score indicates greater acceptability. Diet and physical activity specific versions were administered separately, with items adapted to each context (e.g. ‘support to optimise my diet to treat insomnia symptoms meets my approval’ and ‘I welcome support to optimise my physical activity to treat insomnia symptoms’). Internal consistency was high (Cronbach's *α* = 0.9 for diet; 0.94 for physical activity).

### 2.3. Variable transformations

IPAQ-SF data for vigorous and moderate activity were used to calculate a second physical activity variable reflecting whether participants met minimum physical activity guidelines (≥30 minutes of moderate-intensity activity, ≥5 days a week). Participants reporting any combination of moderate and/or vigorous activity for ≥30 minutes, ≥5 days/week were classified as meeting guidelines. Prior research exploring the links between physical activity patterns and insomnia symptoms highlights the importance of both regularity/frequency [i.e. ≥30 minutes, ≥5 days a week (Li et al., 2024)] and intensity [i.e. at least moderate (Tian et al., 2024)].

As standardised AIM cut-off scores are unavailable, acceptability was defined a priori as mean AIM scores ≥4, consistent with previous lifestyle (Watkins et al., 2024) and sleep research (Mendieta et al., 2025). This threshold corresponds to an average response of ‘agree’ or higher across AIM items and was selected as a conservative approach to avoid overestimating acceptability. The number and percentage of participants meeting the threshold were reported, and a binary acceptability variable was created for the primary regression analyses. This outcome was selected given the aim was to identify participant characteristics associated with meeting the predefined acceptability threshold, rather than characteristics associated with higher AIM scores that may not necessarily indicate overall acceptability.

### 2.4. Data analyses

Descriptive statistics summarised participant clinical and demographic information, MEDAS adherence, physical activity, regularity of meal and physical activity patterns, history of insomnia treatment and acceptability of support for diet and physical activity.

Exploratory multivariable binary logistic regressions examined whether age, gender, education and insomnia severity were associated with Mediterranean diet adherence (MEDAS ≤5, >5), levels of physical activity (<30 minutes of moderate-intensity activity on 5 days/week, ≥30 minutes on ≥5 days/week) and regularity of meal (Yes, No) and physical activity patterns (Yes, No).

Two additional multivariable binary logistic regressions assessed whether the same characteristics were associated with acceptability of receiving diet and physical activity support (AIM <4 [neutral/unacceptable], AIM ≥4 [acceptable]). Sensitivity analyses examined whether findings were consistent when acceptability was modelled continuously using the mean of the four AIM items rather than dichotomised. Separate general linear models were conducted for diet and physical activity acceptability with age, gender, education and insomnia severity entered as categorical fixed factors.

Responses of ‘prefer not to say’ (education), ‘non-binary’ (gender) and ‘unsure’ (meal pattern irregularity) were treated as missing for association analyses due to insufficient numbers. Logistic regression models were assessed for sparse or empty cells, complete or quasi-complete separation, multicollinearity and influential cases, with no issues identified. General linear model assumptions were assessed using residual and Q-Q plots, Levene's test, standardised residuals and Cook's distance, with no substantial violations identified. Each regression model included a single outcome and was adjusted for age, gender, education and insomnia severity. All regressions used the enter method, with *p* < 0.05 considered statistically significant.

### 2.5. Ethics statement

This research was approved by the Human Research Ethics Committee at the University of the Sunshine Coast (approval number: A242037).

## 3. Results

### 3.1. Participants

**Figure 1.**
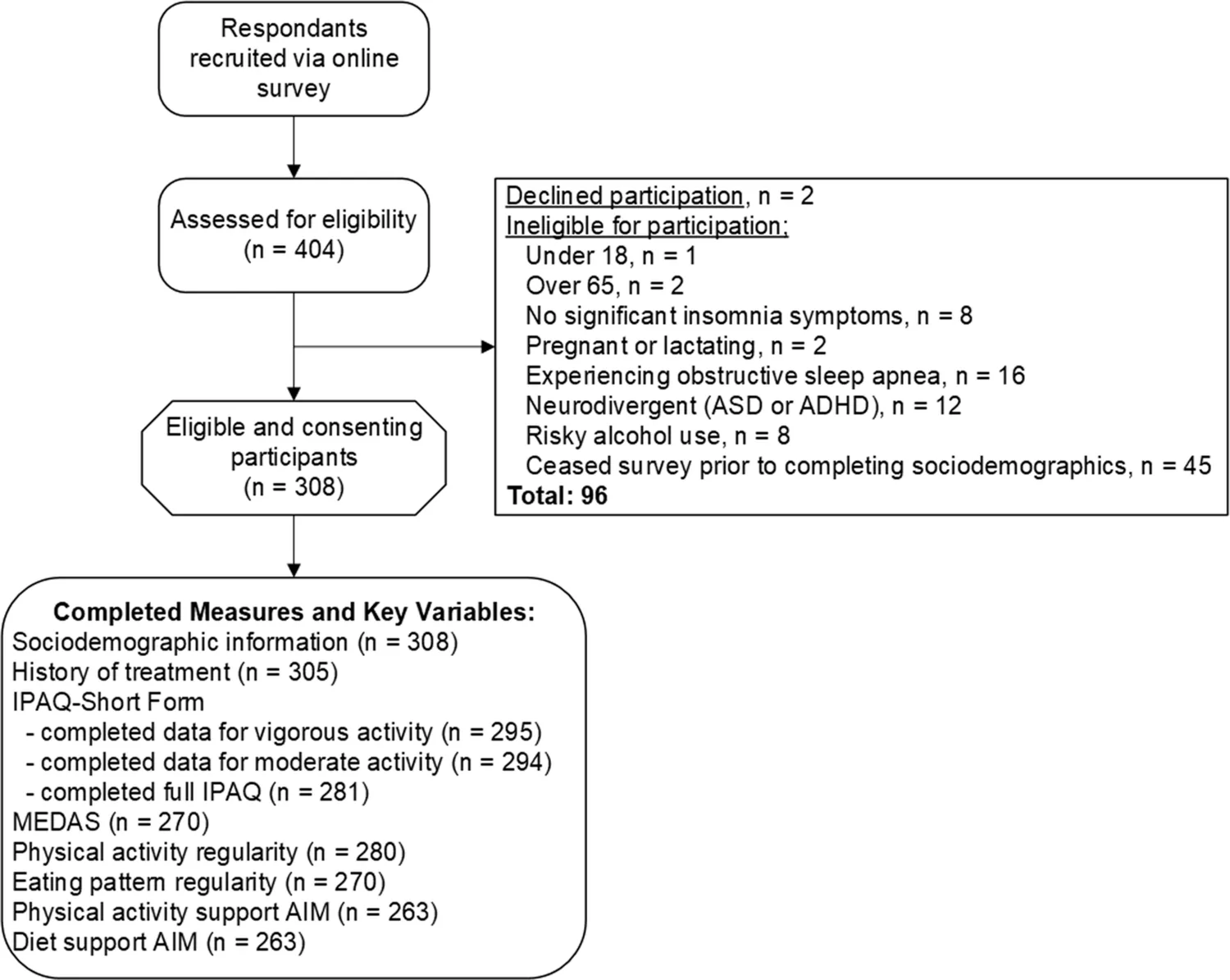
Participant flowchart. Abbreviations: IPAQ = International Physical Activity Questionnaire; MEDAS = Mediterranean Diet Adherence Screener; AIM = Acceptability of Intervention Measure.

Figure 1 outlines the recruited sample. Of the 404 individuals assessed for eligibility, two declined to participate, and 49 were deemed ineligible. A further 45 participants were excluded from analysis because their incomplete survey responses provided no information on sleep, diet, or physical activity. Data from the remaining sample of 308 participants were available for analysis (Figure 1). Approximately half of participants were recruited via Prime Panels (*n* = 151), a small minority by flyers at local universities (*n* = 10) and the remainder of participants were recruited via distribution of recruitment material on social media (*n* = 147).

### 3.2. Demographic and clinical information

The sample of 308 participants was predominantly female (84.7%, *n* = 261), with the majority born in Australia (75%) and speaking English as their primary language (92.5%). Insomnia symptom severity varied from subthreshold (35.7%), to moderate (51.0%) and severe (13.3%). Detailed demographic and clinical characteristics are presented in Tables 1 and 2, respectively.

**Table 1.** Participant demographic information.

Characteristic	Total (*n* = 308)
Gender	*n* (%)
Male	46 (14.9)
Female	261 (84.7)
Non-binary	1 (0.3)
Age (years)	
18–24	35 (11.4)
25–34	64 (20.8)
35–44	55 (17.9)
45–54	66 (21.4)
55–65	88 (28.6)
First Nations person	
No	297 (96.4)
Aboriginal	9 (2.9)
Prefer not to say	2 (0.6)
Country of birth	
Australia	231 (75.0)
United Kingdom	21 (3.8)
Southeast Asia	15 (4.9)
New Zealand	8 (2.6)
Other^a^	32 (10.4%)
Prefer not to say	1 (0.3)
Primary spoken language	
English	285 (92.5)
Cantonese	4 (1.3)
Greek	2 (0.6)
Indonesia	2 (0.6)
Mandarin	2 (0.6)
Portuguese	2 (0.6)
Tagalog	2 (0.6)
Other^b^	8 (2.6)
Prefer not to say	1 (0.3)
Highest education level achieved	
Never attended school	1 (0.3)
Year 9 or below	10 (3.2)
Year 10	22 (7.1)
Year 12 or equivalent	48 (15.6)
Vocational education and training^c^	94 (30.5)
Bachelor's degree	80 (26.0)
Postgraduate degree^d^	52 (16.9)
Prefer not to say	1 (0.3)
Marital status	
Married/in a de facto relationship	142 (46.1)
Never married	112 (36.4)
Divorced/separated	46 (14.9)
Widowed	4 (1.3)
Other	2 (0.6)
Prefer not to say	2 (0.6)
Employment status	
Full-time paid employment	108 (35.1)
Part-time/casual paid employment	88 (28.6)
Full-time parent/caregiver	22 (7.1)
Unemployed	25 (8.1)
Student/unpaid traineeship	17 (5.5)
Volunteer	6 (1.9)
Retired	16 (5.2)
Unable to work due to a disability	16 (5.2)
Other	10 (3.2)

^a^ Includes *n* < 5 from the following countries: Bolivia, Brazil, Canada, China, Cyprus, Fiji, France, Germany, Hong Kong, India, Iran, Italy, Libya, Papua New Guinea, Serbia, South Africa, South Korea, United States, Zimbabwe.

^b^ Includes *n* = 1 for each of the following languages: Bengali, Italian, Korean, Persian, Serbian, Spanish, Tamil and Vietnamese.

^c^ Includes the following qualifications: Trade/pprenticeship, Certificate I/II/III/IV or Diploma/Advanced diploma.

^d^ Includes the following qualifications: Graduate certificate, Graduate diploma, Master's degree, PhD.

**Table 2.** Participant clinical information.

Characteristic	Total (*n* = 308)
Insomnia severity index (ISI)	*n* (%)
Subthreshold insomnia	110 (35.7)
Clinical insomnia (moderate)	157 (51.0)
Clinical insomnia (severe)	41 (13.3)
Self-reported general health status, *n* = 306^a^	
Very good	18 (5.9)
Good	89 (29.1)
Relatively good	149 (48.7)
Bad	46 (15.0)
Very bad	4 (1.3)
Diagnosed or treated for mental health disorder^b^, *n* = 306^c^	
No	198 (64.7)
Yes	108 (35.3)
Anxiety-related disorder	80 (26.1)
Obsessive-compulsive disorder	8 (2.6)
Post-traumatic stress disorder	24 (7.8)
Mood disorder	36 (11.8)
Substance-use disorder	2 (0.6)
Somatic symptom disorder	7 (2.3)
Eating disorder	8 (2.6)
Personality disorder	6 (2.0)
Other	3 (0.9)
Prefer not to say	16 (5.2)

^a^ Missing data for self-reported general health status: *n* = 2 (0.6%).

^b^ Diagnosed or treated in the last 12 months, multiple selection allowed.

^c^ Missing data for diagnosis or treatment of mental health disorder: *n* = 2 (0.6%).

### 3.3. Diet, physical activity and regularity of meal and activity patterns

Most participants reported low adherence to the Mediterranean diet (72.2%), with only 1% reporting high adherence and 26% reporting moderate adherence. A majority (68.5%) did not meet the physical activity guidelines of at least 30 minutes of moderate-intesntiy activity on five days per week. Physical activity levels as classified by the IPAQ-SF, which converts self-reported activity into MET-minutes to account for both intensity and duration of activity, showed a distribution across the low (32.2%), moderate (33.7%) and high (34.1%) activity categories. Additionally, irregular meal patterns were common (70.7%), as were irregular physical activity patterns (59.6%). See Table 3 for detailed descriptive statistics. Of those who were physically inactive, 98.9% also had low or moderate adherence to the Mediterranean diet. One person (0.4%) of the entire sample had high adherence to the Mediterranean diet and was also meeting physical activity guidelines.

**Table 3.** Descriptive statistics for insomnia severity index (ISI), Mediterranean diet adherence screener (MEDAS), International Physical Activity Questionnaire Short-form (IPAQ-SF) and regularity of meal and activity patterns among participants with insomnia symptoms.

Variable	*n* (%)	Mean (SD)	Median (range: min–max)
ISI, *n* = 308		16.56 (4.32)	16.00 (8–28)
IPAQ-SF, *n* = 273^a^			
Low	88 (32.2)		
Moderate	92 (33.7)		
High	93 (34.1)		
Meeting physical activity guidelines, *n* = 292			
Yes	92 (31.5)		
No	200 (68.5)		
MEDAS, *n* = 270		4.34 (1.82)	4.00 (0–9)
Low (≤5)	195 (72.2)		
Moderate (6–8)	72 (26.7)		
High (≥9)	3 (1.0)		
Regular meal patterns, *n* = 270			
Yes	76 (28.1)		
No	191 (70.7)		
Unsure	3 (1.0)		
Regular activity patterns, *n* = 280			
Yes	113 (40.5)		
No	167 (59.6)		

Note: *n* varies between items due to the inclusion of partial responses. MEDAS: Mediterranean Diet Adherence Screener; IPAQ: International Physical Activity Questionnaire.

^a^ *n *= 8 responded ‘don't know’ to amount of activity across items; these were treated as missing data for the purpose of data analysis.

### 3.4. Associations between Mediterranean diet adherence, physical activity and regularity of meal and activity patterns with key participant characteristics

#### 3.4.1. Diet

There were significant associations between key participant characteristics and adherence to the Mediterranean diet. The overall adjusted model, which included age, gender, education and insomnia severity, was statistically significant, χ^2^ (df = 10, *N* = 268) = 44.59, *p* < .001, and explained 22% of the variance (Nagelkerke *R* ^2^ = 0.22). Males had higher odds of low adherence to the Mediterranean diet compared to females (odds ratio [OR] = 4.67, 95% confidence interval [CI]: 1.61–13.31, *p* = .004). Compared with participants aged 55–65 years, those aged 18–24, 25–34 and 35–44 years had 4.3–6.1 times higher odds of low adherence (see Table 4; all *p*s < .05). Relative to Year 12 or below, vocational education and training (70% lower odds; 95% CI: 0.12–0.76, *p* = .01), bachelor's degree (68% lower odds; 95% CI: 0.12–0.86, *p* < .03) and postgraduate degree (85% lower odds; 95% CI: 0.05–0.43, *p* < .01) were associated with reduced odds of low adherence.

#### 3.4.2. Physical activity

Key participant characteristics were not reliably associated with the likelihood of adherence to physical activity guidelines, as the overall logistic regression model was not statistically significant, χ^2^ (*df* = 10, *N* = 290) = 10.60, *p* = .390 (Table 4).

#### 3.4.3. Irregular meal patterns

There was an association between key participant characteristics and irregular meal patterns in the model, which was adjusted for age, gender, insomnia severity and education. The overall model was significant, χ^2^ (df = 10, *N* = 265) = 34.92, *p* < .001, and explained 17% of the variance (Nagelkerke *R* ^2^ = 0.17). Compared to those aged 55-65 years, those aged 18–24, 25–34, 35–44 and 45–54 years were 15.7 (95% CI: 3.25–76.03, *p* < .01), 2.8 (95% CI: 1.28–6.31, *p* = .01), 3.4 (95% CI: 1.44–8.27, *p* < .01) and 2.6 (95% CI: 1.17–5.86, *p* = .01) times the odds, respectively, of having an irregular meal pattern (Table 4). Individuals with a vocational education or a bachelor's degree had 2.4 (95% CI: 1.10–5.25, *p* < .03) and 2.9 (95% CI: 1.19–7.06, *p* < .02) higher odds, respectively, of reporting irregular meal patterns compared to those with a Year 12 education or below (see Table 4).

#### 3.4.4. Irregular physical activity

The overall model was not statistically significant, χ^2^ (*df* = 10, *N* = 278) = 14.50, *p* = 0.152 (Table 4), indicating that age, gender, insomnia severity and education were not reliably associated with regularity of physical activity patterns.

**Table 4.** Multivariable associations between low adherence to the Mediterranean diet, not meeting physical activity guidelines, irregular meal patterns and irregular physical activity with key participant characteristics.

		Low adherence to the Mediterranean diet^a^		Not meeting physical activity guidelines^b^		Irregular meal patterns^c^		Irregular physical activity^d^	
Variable	Category	OR (95% CI)	*p*-value	OR (95% CI)	*p*-value	OR (95% CI)	*p*-value	OR (95% CI)	*p*-value
Gender	Female	1.00		1.00		1.00		1.00	
	Male	4.67 (1.63–13.32)	.004*	0.92 (0.46–1.86)	.824	1.31 (0.60–2.89)	.500	1.18 (0.58–2.39)	.646
Age (years)	55–65	1.00		1.00		1.00		1.00	
	18–24	6.10 (1.57–23.73)	.009*	0.75 (0.30–1.89)	.539	15.71 (3.25–76.03)	<.001*	0.93 (0.35–2.44)	.930
	25–34	4.32 (1.81–10.35)	.001*	0.70 (0.33–1.50)	.360	2.85 (1.28–6.31)	.010*	0.64 (0.31–1.33)	.642
	35–44	5.68 (2.18–14.82)	<.001*	0.47 (0.22–1.03)	.059	3.45 (1.44–8.27)	.005*	0.76 (0.35–1.62)	.477
	45–54	2.27 (1.00–5.17)	.051	1.07 (0.49–2.31)	.871	2.62 (1.17–5.86)	.019*	1.04 (0.50–2.17)	.910
Insomnia Severity Index (ISI)	Subthreshold insomnia	1.00		1.00		1.00		1.00	
	Clinical insomnia (moderate severity)	0.96 (0.50–1.85)	.908	1.43 (0.65–3.17)	.373	0.76 (0.40–1.44)	.395	0.72 (0.42–1.24)	.232
	Clinical insomnia (severe)	2.00 (0.70–5.50)	.197	1.17 (0.54–2.52)	.688	1.50 (0.55–3.87)	.412	0.87 (0.39–1.94)	.731
Education	Year 12 or below	1.00		1.00		1.00		1.00	
	Vocational education and training	0.30 (0.12–0.76)	.011*	1.10 (0.55–2.19)	.788	2.41 (1.10–5.25)	.027*	0.48 (0.23–0.99)	.046
	Bachelor's degree	0.32 (0.12–0.86)	.025*	1.26 (0.61–2.60)	.533	2.91 (1.19–7.06)	.018*	0.39 (0.19–0.82)	.013
	Postgraduate degree	0.15 (0.05–0.43)	<.001*	2.79 (1.11–7.01)	.029	1.26 (0.52–3.05)	.613	0.49 (0.21–1.13)	.093

Note: *p*-values marked with an asterisk are significant (*p* < .05) and are from regressions where the model's omnibus (overall) test was significant (*p* < .05). Estimates are adjusted for age, gender, education and insomnia severity. Abbreviations: OR = Odds Ratio; CI = confidence interval. Responses of ‘prefer not to say’ for education (*n* = 1), ‘unsure’ for irregular eating patterns (*n* = 3) and ‘non-binary’ for gender (*n* = 1) were treated as missing data due to insufficient numbers for analysis. Pairwise deletion was used for each analysis.

^a^ *n* = 268, missing data = 2 (0.7%). Low adherence to the Mediterranean diet represents participants who scored ≤ 5 on the Mediterranean Diet Adherence Screener.

^b^ *n* = 290, missing data = 2 (0.7%). Not meeting physical activity guidelines represents participants who did not engage in a minimum of 30 minutes of moderate-to-vigorous intensity physical activity on 5 or more days per week.

^c^ *n* = 265, missing data = 5 (1.9%).

^d^ *n* = 278, missing data = 2 (0.7%).

### 3.5. History of insomnia treatment

Of the respondents, 19% (*n* = 58) had previously received insomnia treatment from a health professional (Supplementary Table 1), with the majority having received treatment from a GP (78.2%). The most common form of treatment was medication (67.3%), followed by general counselling (34.5%) and CBT-I (20%). Although some participants reported that the treatment they received made brief reference to the impact of diet (23.6%) and physical activity (43.6%) on sleep, few participants received more structured support to make changes to diet (3.6%) or physical activity (7.3%).

### 3.6. Acceptability of receiving support to optimise diet and physical activity in the treatment of insomnia

Overall, rates of acceptability for receiving support to optimise diet and physical activity were similar, with the majority (57%) of participants finding support for both diet and physical activity acceptable (mean AIM = 3.8, SD = 0.8, Table 5). Of those who had low adherence to the Mediterranean diet, 52.6% found support to change their diet acceptable, compared with 70% of those with moderate adherence (not in table). Of those who were not meeting physical activity guidelines, 54.4% found support to change their physical activity acceptable.

**Table 5.** Acceptability of receiving support to optimise diet and physical activity in the treatment of Insomnia.

	Mean (SD)	Median (range: min–max)	*n* (%)
Acceptability of receiving support to optimise diet in insomnia treatment, *n* = 263			
Acceptability (AIM) of receiving support (overall)	3.84 (0.76)	4.00 (1–5)	
* Item 1. Support meets my approval*	3.72 (0.87)	4.00 (1–5)	
* Item 2. Support would be appealing to me*	3.86 (0.88)	4.00 (1–5)	
* Item 3. I would like support*	3.86 (0.87)	4.00 (1–5)	
* Item 4. I welcome support*	3.90 (0.85)	4.00 (1–5)	
Respondents with AIM score ≥4			150 (57.0)
Acceptability of receiving support to optimise physical activity in insomnia treatment, *n* = 263			
Acceptability (AIM) of receiving support (overall)	3.82 (0.83)	4.00 (1–5)	
* Item 1. Support meets my approval*	3.78 (0.90)	4.00 (1–5)	
* Item 2. Support would be appealing to me*	3.84 (0.90)	4.00 (1–5)	
* Item 3. I would like support*	3.81 (0.92)	4.00 (1–5)	
* Item 4. I welcome support*	3.84 (0.90)	4.00 (1–5)	
Respondents with AIM score ≥4			151 (57.4)

Note: AIM = Acceptability of Intervention Measure.

### 3.7. Association between acceptability of receiving support for optimising diet and physical activity with gender, age, insomnia severity and education

#### 3.7.1. Acceptability of support for optimising diet

The binary logistic regression model was statistically significant, χ^2^ (*df* = 10, *N* = 261) = 39.94, *p* < .001, and explained 19% of the variance in acceptability (Nagelkerke *R* ^2^ = 0.19). Females had 2.7 times the odds of finding diet support acceptable compared to males (95% CI: 1.32–5.71, *p* < .01), while participants with severe insomnia had 8.4 (95% CI: 2.65–26.32, *p* < .01) times higher odds of finding it acceptable than those with subthreshold insomnia. There was also an increase in acceptability with those aged 45–54 (OR = 4.0, 95% CI: 1.39–11.7, *p* = .01) compared to those aged 18–24 (Table 6). The sensitivity analysis supported the findings for gender and insomnia severity; however, age was not associated with acceptability when AIM scores were modelled continuously. Differences in acceptability related to age should be interpreted with caution.

#### 3.7.2. Acceptability of support for optimising physical activity

Key participant characteristics predicted whether physical activity support was acceptable. The binary logistic regression model was statistically significant, χ^2^ (*df* = 10, *N* = 261) = 24.35, *p* = .004, and 12% of the variance was explained by the model (Nagelkerke *R* ^2^ = 0.12). Females had 2.1 times the odds of finding physical activity support acceptable compared with males (95% CI: 1.06–4.33, *p* < .04), and participants with severe insomnia had 4.8 times the odds of finding it acceptable compared with those with subthreshold insomnia (95% CI: 1.78–13.07, *p* < .01; Table 6). The sensitivity analysis supported these findings.

**Table 6.** Multivariable associations between acceptability (AIM) of receiving support for optimising diet and physical activity in the treatment of insomnia, and key participant characteristics.

		Diet support acceptability		Physical activity support acceptability	
Variable	Category	OR (95% CI)	*p-*value	OR (95% CI)	*p-*value
Gender	Male	1.00		1.00	
	Female	2.74 (1.32–5.71)	.007*	2.14 (1.06–4.33)	.035*
Age (years)	18–24	1.00		1.00	
	25–34	2.27 (0.82–6.27)	.115	0.90 (0.34–2.40)	.836
	35–44	1.70 (0.56–4.91)	.325	0.89 (0.32–2.47)	.815
	45–54	4.00 (1.39–11.7)	.010*	1.48 (0.54–4.10)	.447
	55–65	2.28 (0.84–6.22)	.106	0.79 (0.30–2.07)	.636
Insomnia Severity Index (ISI)	Subthreshold insomnia	1.00		1.00	
	Clinical insomnia (moderate severity)	1.26 (0.72–2.21)	.422	1.12 (0.64–1.95)	.689
	Clinical insomnia (severe)	8.35 (2.65–26.32)	<.001*	4.83 (1.78–13.07)	.002*
Education	Year 12 or below	1.00		1.00	
	Vocational education and training	0.89 (0.43–1.86)	.766	1.14 (0.56–2.33)	.714
	Bachelor's degree	1.48 (0.68–3.20)	.323	1.72 (0.81–3.66)	.157
	Postgraduate degree	2.19 (0.89–5.39)	.088	2.03 (0.85–4.82)	.109

Note: *n* = 261. *P*-values marked with an asterisk are significant (*p* < .05) and are from regressions where the model's omnibus (overall) test was significant (*p* < .05). Abbreviations: OR = Odds Ratio; CI = Confidence Interval. Responses of ‘prefer not to say’ for education (0.3%, *n* = 1) and ‘non-binary’ for gender (0.3%, *n* = 1) were treated as missing data due to insufficient numbers for analysis. Total missing data was 0.6% (*n* = 2). Pairwise deletion was used.

## 4. Discussion

### 4.1. Overview

This is the first study internationally to comprehensively characterise diet and physical activity among people with insomnia and explore the acceptability of receiving support to optimise these behaviours as part of routine treatment. Findings indicate that most of the sample may have an unmet need for support to address diet and physical activity behaviours, specifically to increase adherence to the Mediterranean diet and physical activity guidelines, as well as the regularity of eating and activity. Additionally, findings suggest men, younger adults, and those with lower levels of education may be important groups to consider when developing interventions to support these behaviours. Overall, 57% of the sample endorsed that they would find receiving support to address diet and physical activity as part of insomnia treatment highly acceptable. Acceptability of such support was highest among women and those with severe symptoms. Understanding these behaviours along with the views of those with insomnia is a crucial first step to the development of a robust and meaningful lifestyle intervention that may support diet and physical activity behaviour change.

### 4.2. Adherence and regularity of diet and physical activity behaviours

The current study identifies Mediterranean diet adherence as a potentially important intervention target for adults with insomnia symptoms, given 72% of the sample reported low adherence and only 1% reported high adherence. These findings may indicate an unmet need for dietary support, particularly as people with insomnia symptoms may be at greater risk of poorer diet quality than the general population. While nationally representative Australian MEDAS data are limited, available Australian community samples report mean scores between 5.2 and 7.2 (out of 14) (Byrne-Kirk et al., 2024; Kabthymer and Danaher, 2025). In contrast, the current sample scored 4.3 (SD 1.8), suggesting lower adherence to the Mediterranean diet.

Lower adherence to the Mediterranean diet was associated with lower education, younger age, and male gender. These education and age-related differences have been previously observed in general populations, with lower levels of eduction and younger age associated with lower adherence (Mendonça et al., 2022). One proposed explanation is that older adults may have greater routine stability and health awareness (Ammar et al., 2025). Consistent with this, younger participants in the current study were more likely to report irregular meal patterns. Higher education may also support healthier choices through greater health literacy (Azizi Fard et al., 2021). However, participants with vocational training and bachelor's level education were more likely to report irregular meal patterns, suggesting education-linked factors (e.g. occupational factors such as shift work) may disrupt meal regularity. While dietary patterns in insomnia have been examined, day-to-day meal regularity and its sociodemographic correlates remain underexplored. Evidence for gender differences in Mediterranean diet adherence is heterogeneous (Brandt et al., 2025), and little work has examined these associations specifically in insomnia samples. In the general population, evidence suggests men are less likely to engage in preventative healthcare (Hiller et al., 2017), which may contribute to the poorer diet reported in the current sample. However, men comprised only 15% of this sample, so findings concerning gender-related differences are primarily exploratory. Overall, younger adults, men, and those with lower education may represent important groups to consider when developing interventions to increase adherence to the Mediterranean diet. Importantly, as very few participants had high adherence to the Mediterranean diet, data from this study indicate a potential unmet need for dietary support across the broader cohort of people with insomnia symptoms.

Physical activity levels were also low, as only 32% of participants achieved at least 30 minutes of moderate-intensity activity on five days per week – the minimum recommended for physical activity interventions to improve insomnia (Riedel et al., 2024). This suggests that nearly 70% may have an unmet need for support to increase physical activity. This proportion is also substantially lower than population-level estimates. In the 2022 Australian Bureau of Statistics National Health Survey, 56% of Australian adults reported completing 30 minutes of activity on five or more days (Australian Bureau of Statistics, 2022). Together, these findings suggest adults with insomnia symptoms may be at an increased risk of physical inactivity. No clear subgroup differences emerged for meeting physical activity guidelines or maintaining regular activity patterns, suggesting physical activity may be an important intervention target across individuals with insomnia, regardless of key sociodemographic or clinical characteristics.

Meal and physical activity patterns were often irregular (71% and 60%, respectively), indicating a potential benefit from interventions that improve the regularity of these behaviours. Aligning food intake with the circadian rhythm (‘chrononutrition’) is increasingly implicated in sleep-disorder treatments and may improve sleep quality, although long-term efficacy remains uncertain (Patel & Cheung, 2025). Similarly, the timing of physical activity can influence circadian rhythm (Haupt et al., 2021) and is an emerging therapeutic focus for sleep disorders (Lee et al., 2024). Given that eating and physical activity can act as circadian zeitgebers, further research is needed to explore whether interventions targeting regularity reduce circadian misalignment and support sleep outcomes among people with insomnia symptoms.

### 4.3. Acceptability of receiving support to address poor diet and physical inactivity

Treatments targeting lifestyle risk behaviours may be well positioned to succeed within this population, with 57% of participants on average *agreeing* or *completely agreeing* that receiving support to optimise both diet and physical activity as part of insomnia treatment would be acceptable (mean AIM = 3.8, SD 0.8). Within the broader behavioural and psychological intervention development field, acceptability is conceptualised as multi-faceted and may vary within target populations rather than being unanimous (Sekhon et al., 2017), with mean AIM scores above 3.5 commonly considered to be indicative of acceptability (Koch et al., 2025; McLaughlin et al., 2024). While findings from the current study suggest receiving support to optimise diet and physical activity would be an acceptable part of insomnia treatment, they also indicate that this is not happening routinely. Less than half of participants who had received treatment reported that their treatment mentioned diet (24%) or physical activity (44%), and very few (4%–7%) received support to actually implement and sustain changes.

Participants with severe insomnia symptoms were more likely to consider support for optimising diet (OR = 8.4) and physical activity (OR = 4.8) acceptable. Greater symptom burden may increase perceived need for treatment and willingness to try novel approaches, whereas those with moderate or subthreshold symptoms may view their sleep difficulties as manageable without formal support. This aligns with evidence that preferences for behavioural treatments are strongest among people with severe insomnia symptoms (Perez et al., 2022). Previous treatment experiences may also shape subsequent perceptions and preferences, particularly when earlier approaches have provided insufficient benefit (Sidani et al., 2019). People with more severe insomnia symptoms may have tried a greater number of treatments with varying levels of success and may therefore be more open to novel approaches.

Middle-aged adults (aged 45–54) appeared more likely to meet the predefined acceptability threshold for dietary support, although this association was not observed when AIM scores were modelled continuously. This suggests differences may relate to the likelihood of crossing the predefined threshold, rather than to higher overall acceptability. Further research is needed to determine whether acceptability varies by age.

Just as women were more likely to adhere to the Mediterranean diet, findings suggest they were also more likely than men to view support for improving diet and physical activity as acceptable. Gender-related differences have been identified in both the prevalence (Benjafield et al., 2025) and the management of insomnia (Sidani et al., 2019). Women report using a wider range of insomnia management strategies, including complementary approaches, whereas men may be more likely to minimise symptoms or endorse unhelpful beliefs about insomnia (Sidani et al., 2019). Differences in acceptability may reflect broader gendered patterns in the management of insomnia and health concerns. Women have been found to engage more actively with health information (Tong et al., 2014) and to hold more favourable attitudes towards seeking professional psychological support (Nam et al., 2010). Gendered social norms surrounding health care and help-seeking may partially explain the observed differences in acceptability. Future lifestyle interventions should consider how men can be appropriately engaged to ensure that differences in treatment acceptability do not contribute to inequities in engagement with insomnia care.

### 4.4. Limitations and generalisability

Firstly, the sample appears broadly representative of age and gender trends reported in epidemiological studies of insomnia (Benjafield et al., 2025) and reflects major population centres, with most respondents from New South Wales (26.6%), Queensland (31.2%) and Victoria (26.0%). However, findings may not generalise to rural and remote communities where health support is more limited. Secondly, despite the sample being reflective of gender distribution trends, the small number of men and gender-diverse participants limits the generalisability of the findings to these groups. Observed gender differences should therefore be considered exploratory. Further studies should recruit more men and gender-diverse participants to better understand whether lifestyle behaviours and intervention preferences vary by gender. Thirdly, this study focused on the Mediterranean diet due to its strong links with insomnia symptom reduction. Further research might consider other approaches to categorising healthy dietary patterns where epidemiological research indicates there could be associations with insomnia risk and symptom reduction. Fourthly, including partial responses may have introduced response bias. Partial completion appeared slightly more common among participants with moderate or severe insomnia, meaning later survey measures, such as acceptability, may underrepresent those with greater symptom severity. Finally, diet, physical activity, and symptoms of obstructive sleep apnoea were assessed through self-report. Recall bias and social desirability bias may have resulted in the overestimation of Mediterranean diet adherence and physical activity, while undiagnosed or misreported sleep disorders may have been present. Future research into lifestyle behaviours among those with insomnia should consider the use of objective or other validated measures. Despite these limitations, the study provides insights into diet and physical activity behaviours among people with insomnia symptoms and the acceptability of lifestyle-related support, while also identifying areas for future research.

### 4.5. Conclusion

Overall, findings indicate a potential unmet need for structured support to optimise diet and/or physical activity, and many participants considered such support acceptable. Treatments to optimise diet and physical activity may focus on increasing adherence to the Mediterranean diet and physical activity guidelines, but should also consider the timing of these behaviours given associations with reducing insomnia symptoms and supporting sleep-related outcomes. Population subgroups with the lowest adherence to the Mediterranean diet and physical activity guidelines, and those less likely to find receiving support acceptable, need to be considered during intervention development and dissemination. Future research should adopt a formal intervention-development process that addresses the unique determinants of lifestyle behaviour change for people with insomnia. This should include identifying barriers and facilitators to changing diet and physical activity behaviours, determining appropriate behaviour-change strategies, and exploring whether support is best integrated into CBT-I or delivered as an adjunct. Engagement with consumers and key stakeholders could be considered to co-design content and delivery approaches.

## Supplementary Material

SupplementaryFile1.xlsx

## Data Availability

The data that support the findings of this study are available on request from the corresponding author, [JH]. The data are not publicly available due to privacy and ethical restrictions.
